# Prediction of Body Mass Index Using Concurrently Self-Reported or Previously Measured Height and Weight

**DOI:** 10.1371/journal.pone.0167288

**Published:** 2016-11-29

**Authors:** Zhaohui Cui, June Stevens, Kimberly P. Truesdale, Donglin Zeng, Simone French, Penny Gordon-Larsen

**Affiliations:** 1 Department of Nutrition, Gillings School of Global Public Health, University of North Carolina at Chapel Hill, Chapel Hill, North Carolina, United States of America; 2 Department of Epidemiology, Gillings School of Global Public Health, University of North Carolina at Chapel Hill, Chapel Hill, North Carolina, United States of America; 3 Department of Biostatistics, Gillings School of Global Public Health, University of North Carolina at Chapel Hill, Chapel Hill, North Carolina, United States of America; 4 Division of Epidemiology and Community Health, School of Public Health, University of Minnesota, Minneapolis, Minnesota, United States of America; University of Tennessee Health Science Center, UNITED STATES

## Abstract

**Objective:**

To compare alternative models for the imputation of BMI_M_ (measured weight in kilograms/measured height in meters squared) in a longitudinal study.

**Methods:**

We used data from 11,008 adults examined at wave III (2001–2002) and wave IV (2007–2008) in the National Longitudinal Study of Adolescent to Adult Health. Participants were asked their height and weight before being measured. Equations to predict wave IV BMI_M_ were developed in an 80% random subsample and evaluated in the remaining participants. The validity of models that included BMI constructed from previously measured height and weight (BMI_PM_) was compared to the validity of models that used BMI calculated from concurrently self-reported height and weight (BMI_SR_). The usefulness of including demographics and perceived weight category in those models was also examined.

**Results:**

The model that used BMI_SR_, compared to BMI_PM_, as the only variable produced a larger R^2^ (0.913 vs. 0.693), a smaller root mean square error (2.07 vs. 3.90 kg/m^2^) and a lower bias between normal-weight participants and those with obesity (0.98 vs. 4.24 kg/m^2^). The performance of the model containing BMI_SR_ alone was not substantially improved by the addition of demographics, perceived weight category or BMI_PM_.

**Conclusions:**

Our work is the first to show that concurrent self-reports of height and weight may be more useful than previously measured height and weight for imputation of missing BMI_M_ when the time interval between measures is relatively long. Other time frames and alternatives to in-person collection of self-reported data need to be examined.

## Introduction

Longitudinal studies of body weight in humans almost always have missing values due to loss to follow-up or incomplete examinations. These missing values can bias estimates and reduce statistical power. A recent review of pharmaceutical randomized trials with weight as the primary outcome indicated that in most studies missing data for body mass index (BMI) was imputed by carrying forward BMI calculated from previously measured height and weight (referred as BMI_PM_) or by predicting the missing values using a model that included BMI_PM_ [1]. However, BMI can change importantly over time, and in some cases those changes are the primary interest of the study. It is well established that BMI calculated from self-reported height and weight (BMI_SR_) is highly correlated with concurrently assessed BMI calculated from measured height and weight (BMI_M_) with r values of approximately 0.90 [2]. Nevertheless, BMI_SR_ tends to underestimate BMI_M_, and this bias is exaggerated in individuals who are female, white, older and heavier [3–6]. Thus both BMI_PM_ and BMI_SR_ have potential to the imputation of BMI_M_, but both have limitations.

The use of modeling to predict missing data is currently recommended for data missing at random [7]. The use of BMI_SR_ in regression models that impute BMI_M_ has been explored in cross-sectional data [3,8–11]. However, to our knowledge, modeling of missing BMI_M_ values using BMI_SR_ has not been extended to longitudinal data. We know of no study that has compared equations that include BMI_PM_ to equations that include BMI_SR_ for the prediction of BMI_M_. We hypothesized that in longitudinal studies in which the most recently measured weight is several years in the past, a regression model to predict BMI_M_ that includes both BMI_PM_ and BMI_SR_ would perform better (i.e. with greater accuracy) than a model that used only BMI_PM_. In addition, we anticipated that BMI_PM_ would produce stronger predictions than BMI_SR_. Since studies have shown that demographics and perception of weight status are associated with BMI_M_ and misreport of BMI_M_ [4,12], we also tested including demographic and perceived weight status variables in the regression models for prediction of BMI_M_.

## Materials and Methods

### Study population

The National Longitudinal Study of Adolescent to Adult Health (Add Health) [13] was selected to provide data for this analysis because both measured and self-reported height and weight were assessed in the same participants on two occasions. The Add Health study was designed to examine the relationship between social, behavioral and biological factors and health from adolescence to adulthood [13]. Samples of youth were drawn from 80 high schools and 52 middle schools selected to ensure representation of US schools in regard to region of country, urbanicity, school size, school type, and school ethnicity. The core sample is nationally representative of American students in grades 7–12 in the 1994–95 school year. Additional special samples provide enlarged subsamples for studies of ethnicity, disability and sibling and twin relations. We combined the data from the core sample and special samples at each wave in the present study. We chose to construct the sample in this way to include a more diverse participants. The first examination (called Wave I) included 20,745 students aged 11–21 years. Follow-up examinations were conducted in 1996–1997 (wave II), 2001–2002 (wave III) and 2007–2008 (wave IV), respectively. We focused the current work on adults and therefore used data from waves III (n = 15,197, age = 18–26 years) and IV (n = 15,701, age = 24–32 years). Survey procedures have been described elsewhere [14] and were approved by the institutional review board at the University of North Carolina at Chapel Hill. Written informed consent was obtained from all participants.

### Measures

At each wave, participants were asked their height and weight in a home-based interview and were measured after the interview by a trained technician. Height was measured to the nearest 0.5 cm without shoes using a measure tape with a standard triangular headboard attached to a wall. Weight was measured to the nearest 0.1 kg without shoes and in clothing using a Health-o-meter 844KL High Capacity Digital Bathroom Scale (Jarden Corporation; Rye, NY). BMI is calculated as weight in kg divided by the square of height in meters. BMI_PM_ was constructed from measured height and weight from wave III, while BMI_M_ and BMI_SR_ were calculated from data collected at wave IV. In wave IV perceived weight status was obtained by asking the participant “how do you think of yourself in terms of weight?”: 1) very underweight; 2) slightly underweight; 3) about the right weight; 4) slightly overweight; 5) very overweight. Because only a few participants answered very underweight (0.7%), this category was combined with slightly underweight.

Participants reported their date of birth and age was calculated. Time intervals were calculated between examination dates. Race/ethnicity was self-reported by parents and adolescents at wave I and was classified as non-Hispanic white, non-Hispanic black, Hispanic, Asian American or Native American [15].

### Analytic sample

Among the 15,701 participants at wave IV, we excluded those who reported pregnancy at wave III or IV (n = 967); had a part of their body amputated (n = 16); were missing race/ethnicity (n = 32), perceived weight status at wave IV (n = 7), or height or weight at either wave (n = 3,631); reported a weight less than 30 pounds (n = 11); or had either a BMI_M_, BMI_SR_ or BMI_PM_ that was <12 kg/m^2^ or > 60 kg/m^2^ (n = 29). Our final analytic sample included 11,008 participants with weight and height data from both waves.

### Statistical analysis

We constructed a training data sets by random selection of 80% of observations without replacement. The remaining participants formed the test dataset. We developed equations to predict BMI_M_ using general linear models and different combinations of variables. The equations developed in the training dataset were applied to the participants in the test dataset to create a predicted BMI_M_. These predicted values were regressed against BMI_M_ in the test dataset and R^2^ and root mean square error (RMSE) were calculated. To evaluate whether the accuracy of the predicted values differed by BMI category [16] we calculated mean signed difference (MSD) as the mean of the difference between predicted values and BMI_M_. To examine whether the results from the above analyses were by chance, we repeated the above analyses in four different sets of training and test datasets generated by randomly allocating the 11,008 participants and found all results were similar [11]. All analyses were conducted with SAS 9.4 software (SAS Institute, INC., Cary, NC).

## Results

In the analytic sample a little over half of the participants were non-Hispanic whites (Table 1). Mean BMI increased from wave III to wave IV in groups with normal weight, overweight and obesity. On average, participants over-reported their measured height. Normal weight participants over-reported, while participants with overweight or obesity under-reported, their measured weight. This resulted in a relatively accurate estimation of BMI_M_ in the normal weight group but underestimation of BMI_M_ by 0.5 kg/m^2^ in the group with overweight and 1.6 kg/m^2^ in the group with obesity.

**Table 1:** Characteristics of the analytic sample.

Characteristics	Overall*	Normal weight*	Overweight*	With obesity*
Sample size	11 008	3 625	3 333	3 873
Male (%)	48.3	42.6	56.1	48.1
Race-ethnicity (%)				
Non-Hispanic white	56.1	61.5	55.7	51.3
Non-Hispanic black	21.0	17.8	20.3	24.6
Hispanic	15.4	11.5	16.4	18.5
Asian	6.8	8.8	7.0	4.6
Native American	0.7	0.4	0.7	1.0
Age at wave III (years) ^†^	21.9 (1.8)	21.8 (1.8)	22.0 (1.8)	22.0 (1.8)
Body mass index (kg/m^2^) ^†^				
BMI_PM_ (wave III)	26.2 (6.0)	21.7 (2.5)	25.2 (3.3)	31.7 (5.9)
BMI_M_ (wave IV)	28.7 (7.0)	22.3 (1.7)	27.4 (1.4)	36.3 (5.5)
BMI_SR_ (wave IV)	28.0 (6.4)	22.4 (1.9)	26.9 (2.1)	34.7 (5.6)
Height (cm) ^†^				
Measured at wave III	170.8 (10.4)	169.9 (10.3)	171.7 (10.4)	170.9 (10.3)
Measured at wave IV	170.1 (10.1)	169.7 (9.9)	171.1 (10.0)	169.7 (10.3)
Self-reported at wave IV	171.2 (10.5)	170.3 (10.5)	172.1 (10.5)	171.4 (10.5)
Weight (kg) ^†^				
Measured at wave III	76.8 (19.4)	63.0 (10.5)	74.6 (12.6)	92.7 (19.3)
Measured at wave IV	83.3 (22.1)	64.5 (9.4)	80.5 (10.2)	104.7 (19.1)
Self-reported at wave IV	82.3 (21.1)	65.1 (10.0)	79.9 (11.1)	101.9 (18.8)
Perceived weight status at wave IV (%)				
Slightly underweight	8.2	16.9	3.2	2.0
About the right weight	35.3	66.2	34.5	6.9
Slightly overweight	43.4	16.7	58.8	57.2
Very overweight	13.1	0.2	3.5	33.9
Years between the two waves^†^	6.5 (0.3)	6.5 (0.3)	6.5 (0.3)	6.5 (0.3)

BMI_PM_, body mass index constructed from measured height and weight at wave III; BMI_M_, body mass index calculated from measured height and weight at wave IV; BMI_SR_, body mass index constructed from self-reported height and weight at wave IV.

* Obesity status was determined using BMI_M_. Overall included participants who are underweight (n = 177), normal weight (n = 3 625), overweight (n = 3 333) and those with obesity (n = 3 873).

^†^ Values were expressed as mean and standard deviation.

Using BMI_M_ as the dependent variable, nine models with different combinations of independent variables were developed in the training data set. The resulting 9 equations (intercepts and coefficients) were applied to the test dataset to generate predicted estimates of BMI_M_. These estimates were then compared to BMI_M_ in the test data using univariate regression. Table 2 shows the R^2^ and RMSE produced from this analysis. Model 1–1 with BMI_PM_ as the only independent variable produced an R^2^ of 0.693 and an RMSE of 3.90 kg/m^2^. Model 2–1 with BMI_SR_ as the only independent variable produced a larger R^2^ (0.913) and a smaller RMSE (2.07 kg/m^2^) compared to the models 1–1. Performance was not substantially improved by adding BMI_PM_ to the model with BMI_SR_. Addition of demographics and perceived weight status also had little impact. Further, the addition of non-linear forms of BMI and age (i.e., inverse, square and cubic), and the interactions of gender or race/ethnicity with BMI did not increase the adjusted R^2^ by more than 0.01 or reduce bias between normal weight group and the group with obesity by as much 0.1 kg/m^2^. The intercepts and coefficients of the models developed in the whole analytic sample are shown in the S1 Table.

**Table 2:** R^2^ and RMSE from regression* of predicted BMI_M_ against actual BMI_M_ in the test dataset.

Model #	Overall	By sex		By race-ethnicity^†^			
		Male	Female	Non-Hispanic white	Non-Hispanic black	Hispanic	Asian
Sample size	2202	1080	1122	1218	462	347	158
R^2^							
BMI_PM_ model							
1–1	0.693	0.662	0.714	0.687	0.714	0.634	0.778
1–2	0.698	0.665	0.720	0.689	0.714	0.633	0.776
1–3	0.770	0.737	0.792	0.771	0.760	0.735	0.829
BMI_SR_ model							
2–1	0.913	0.883	0.932	0.935	0.872	0.894	0.936
2–2	0.914	0.883	0.933	0.935	0.872	0.895	0.936
2–3	0.915	0.885	0.934	0.936	0.875	0.895	0.937
BMI_PM_ and BMI_SR_ model							
3–1	0.918	0.892	0.934	0.935	0.883	0.901	0.942
3–2	0.918	0.893	0.935	0.935	0.883	0.901	0.942
3–3	0.920	0.894	0.936	0.936	0.886	0.902	0.943
RMSE (kg/m^2^)							
BMI_PM_ model							
1–1	3.90	3.62	4.15	3.77	4.11	4.21	3.16
1–2	3.87	3.61	4.10	3.75	4.10	4.21	3.18
1–3	3.38	3.19	3.54	3.22	3.76	3.58	2.77
BMI_SR_ model							
2–1	2.07	2.13	2.02	1.72	2.75	2.26	1.70
2–2	2.07	2.13	2.01	1.72	2.75	2.26	1.70
2–3	2.05	2.11	1.99	1.71	2.72	2.25	1.69
BMI_PM_ and BMI_SR_ model							
3–1	2.02	2.05	1.99	1.71	2.62	2.19	1.61
3–2	2.01	2.04	1.98	1.71	2.62	2.19	1.61
3–3	1.99	2.03	1.96	1.70	2.59	2.18	1.60

RMSE, Root mean square error; BMI_PM_, body mass index constructed from measured height and weight at wave III; BMI_M_, body mass index constructed from measured height and weight at wave IV; BMI_SR_, body mass index constructed from self-reported height and weight at wave IV.

*Independent variable(s) in each model developed in the training dataset to predict BMI_M_: Model 1–1: BMI_PM_; (adjusted R^2^: 0.673; RMSE: 3.98). Model 1–2: BMI_PM_ + age at wave III + time interval + sex + race; (adjusted R^2^: 0.676; RMSE: 3.96). Model 1–3: BMI_PM_ + age at wave III + time interval + sex + race + perceived weight status at wave IV; (adjusted R^2^: 0.754; RMSE: 3.45). Model 2–1: BMI_SR_; (adjusted R^2^: 0.893; RMSE: 2.28). Model 2–2: BMI_SR_ + age at wave IV + sex + race; (adjusted R^2^: 0.893; RMSE: 2.28). Model 2–3: BMI_SR_ + age at wave IV + sex + race + perceived weight status at wave IV; (adjusted R^2^: 0895; RMSE: 2.26). Model 3–1: BMI_PM_ + BMI_SR_; (adjusted R^2^: 0.898; RMSE: 2.23). Model 3–2: BMI_PM_ + BMI_SR_ + age at wave IV + sex + race; (adjusted R^2^: 0.898; RMSE: 2.22). Model 3–3: BMI_PM_ + BMI_SR_ + age at wave IV + sex + race + perceived weight status at wave IV; (adjusted R^2^: 0.900; RMSE: 2.20).

^†^Results for Native Americans were not shown because of small sample size (n = 17).

**Fig 1:**
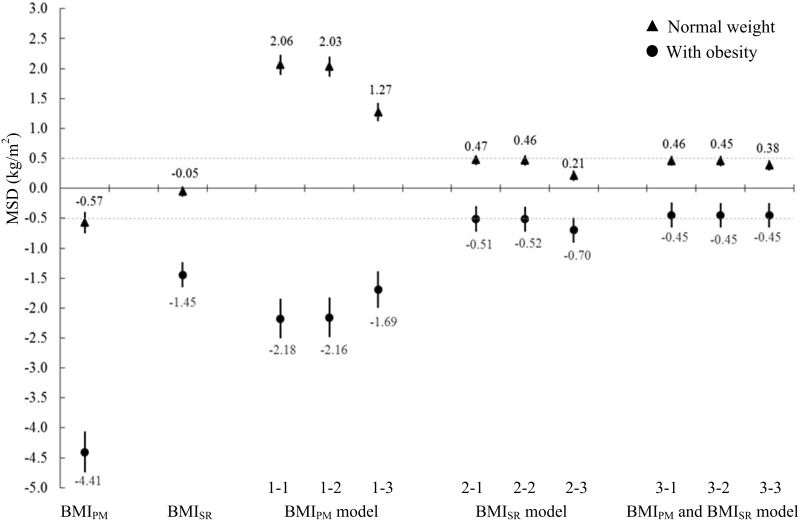
MSD between predicted BMI_M_ and actual BMI_M_ by weight status in the test dataset (n = 2202). (A) Abbreviations: MSD for mean signed difference; BMI for body mass index, BMI_PM_ is derived from measured height and weight at wave III, BMI_M_ is derived from measured height and weight at wave IV, BMI_SR_ is constructed from self-reported height and weight at wave IV. (B) MSD was calculated as the mean of predicted BMI_M_ minus actual BMI_M_. The dashed lines in the Fig are at ±0.5 kg/m^2^. (C) Weight status was based on BMI_M_. n = 719 for normal weight group (18.5≤ BMI_M_ <25 kg/m^2^) and n = 776 for the group with obesity (BMI_M_ ≥30kg/m^2^). Results for underweight group (n = 45) and for overweight group (n = 662) were not shown.

The MSD in overweight adults was -2.52 kg/m^2^ for BMI_PM_, -0.54 kg/m^2^ for BMI_SR_ and close to zero for all nine models (0.03–0.17 kg/m^2^). Those values were intermediate between those seen in the normal weight group and the group with obesity. Fig 1 shows MSD’s calculated using the test dataset that contrast estimates of BMI obtained in normal weight participants and participants with obesity (categories assigned using BMI_M_). The MSD estimates in normal weight participants and participants with obesity were over triple as far from the null for BMI_PM_ compared to BMI_SR_. Model predicted values overestimated BMI_M_ in normal weight participants and underestimated BMI_M_ in participants with obesity with the magnitudes of bias consistently smaller for models that included BMI_SR_ (i.e., models 2–1 to 3–3) compared to models that used BMI_PM_ (i.e., models 1–1, 1–2 and 1–3). As a result, the amount of differential bias (discrepancy between the MSD in normal weight participants and the MSD in participants with obesity for a given model) was lower in models that included BMI_SR_ (i.e., models 2–1 to 3–3). In those models, the range of the discrepancies was relatively narrow ranging from 0.83 to 0.98 kg/m^2^. Analyses conducted by gender and race-ethnicity subgroups also showed smaller discrepancies in the systematic error of estimates produced from models that included BMI_SR_ compared to those that included BMI_PM_.

## Discussion

Our analyses found that equations produced from regression models that included BMI_SR_ performed better than equations that used BMI_PM_. This is consistent with the stronger correlation of BMI_M_ with BMI_SR_ compared to BMI_PM_ in this study with an average of 6.5 years of interval between the two examinations. We found that BMI_SR_ (r = 0.95) was more strongly correlated with BMI_M_ than BMI_PM_ (r = 0.82). Over 100 studies have compared self-reported height and weight with measured values [4–6,17]. Generally these studies have found that adults over-report their height and under-report their weight, resulting in an underestimation of BMI. Despite this bias, BMI_SR_ is very strongly correlated (r >0.90) with concurrent BMI_M_ in most studies [18,19]. BMI_PM_ would be highly correlated with BMI_M_ if weight changes were proportionately similar across individuals over time. However, as shown in the Coronary Artery Risk Development in Young Adults (CARDIA) study weight changes are heterogeneous over a 5-year interval [20].

Only a handful of studies have developed equations to predict BMI_M_ using BMI_SR_ and reported model fit statistics [6,9,21,22]. All those studies used cross-sectional data and therefore did not examine models that used BMI_PM_ as in our present work. Most of those studies stratified their analyses by sex and reported R^2^ ranging from 0.85–0.92 for men and 0.83–0.96 for women [9,21,22], which is consistent with our sex-specific findings. Stommel and Schoenborn [6] developed prediction equations in men and women combined using the 2001–2006 National Health and Nutrition Examination Survey (NHANES) and found an R^2^ of 0.922, which is consistent with our sex-combined results.

We emphasized bias between normal weight participants and participants with obesity in imputation of BMI_M_ because this bias could result in an overestimation of disease risk associated with BMI when predicted values are used in study of BMI-disease relationship [21]. Using data from the NHANES II, Plankey et al. [11] found that predicted values from a model that used BMI_SR_ to predict BMI_M_ tended to increase bias in normal weight adults and reduce bias in adults with overweight or obesity, resulting in an overestimate of BMI_M_ in the normal weight and an underestimate in participants with obesity. In addition to this study, another study is consistent with our work. Using data from the 2001–2006 NHANES, Stommel and Schoenborn [6] also found the predicted values systematically overestimate BMI in leaner individuals and underestimate BMI in heavier individuals. The BMI_M_ at which the predicted BMI is unbiased is 28.3 kg/m^2^.

Although demographics and perceived weight status are associated with misestimation of BMI_M_ [3,4,6,21], we found that addition of those variables and their more complex forms only minimally improved prediction over models that included BMI_SR_ as the only variable. Age was not statistically significant in models that used BMI_SR_, perhaps because of the narrow age range in our sample. However, despite a wider age of participants (30–64 years), Plankey et al. [11] found that addition of age and nonlinear forms of BMI_SR_ (i.e., quadratic and cubic) to a model containing BMI_SR_ alone only slightly improved prediction of BMI_M_ (R^2^: 0.94 vs. 0.94 in women and 0.90 vs. 0.90 in men; RMSE: 1.37 vs. 1.36 in women and 1.22 vs. 1.22 in men). The authors concluded that the bias differed little between the simple and more complex models. Connor Gorber et al. [21] analyzed data from individuals aged ≥18 years who participated in the 2005 Canadian Community Health Survey. They developed a simple model and a full model in the training dataset. The simple model contained BMI_SR_ only and the full model additionally included variables that were significantly associated with the bias in BMI (i.e., age, education, dissatisfied with life and perceived weight status). The R^2^ from the full model is only 0.01 larger than the R^2^ from the simple model. Application of those equations to the test dataset found that the MSDs between BMI predicted by the models and BMI_M_ are close to zero. The full model produced little advantage in the sensitivity and specificity over the simple model in determination of obesity. Similar results were also found in studies from Sweden [9] and Australia [22]. Again, those studies did not use longitudinal data as in our work presented here.

It is important that in our work, measurements of height and weight were collected over a relatively long time interval (6.5 years). Our results are likely not applicable to longitudinal studies with short time intervals between examinations. In addition, participants in our study could have anticipated that they would be measured after reporting their height and weight and this could have reduced bias in the reported data [23]. If investigators collect self-reported height and weight from participants separately from other study variables it could also impact accuracy [23]. Finally, bias would likely have been different if self-reported weight and height had been collected in telephone interviews [10] or mailed survey [24] rather than in-person interviews.

Despite all these issues, our study does provide new information that is relevant to researchers who study participants over relatively long time intervals. In those instances collection of self-reported height and weight could be considered and used with an appropriate approach (e.g., multiple imputation) to improve the imputation of missing BMI_M_. Our work also indicates that investigators should be cautious about relying on previously measured height and weight to indicate current BMI_M_. Since almost all obesity-related longitudinal studies collect demographic variables, those variables could be included in the imputation model, but it appears they improve prediction only very slightly. Perceived weight status was not helpful for imputation of BMI derived from anthropometric measures. Further study is needed to understand time intervals and circumstances in which self-report of height and weight is superior to the other.

## Supporting Information

S1 Table Final models developed to predict BMI_M_ in the whole dataset (n = 11,008). (DOCX)
