# Supplementary material for: Prediction of Body Mass Index Using Concurrently Self-Reported or Previously Measured Height and Weight
Source: PLoS One. 2016 Nov 29;11(11):e0167288. doi: 10.1371/journal.pone.0167288 (PMC5127553; doi:10.1371/journal.pone.0167288)
Supplement: S1 Table — (DOCX) [file pone.0167288.s001.docx]

**Data Availability Statement:** The data were from a third party. The authors are not allowed to release the data according to the Data Use Agreement. To obtain the data, please contact [addhealth@unc.edu](mailto:addhealth@unc.edu).
